# Efficacy and safety of Tuina for treatment of pediatric recurrent respiratory tract infections: A protocol for systematic review and meta-analysis

**DOI:** 10.1097/MD.0000000000027939

**Published:** 2021-12-17

**Authors:** Ye Tian, Lie Wang, Zhongtian Wang, Lizhong Ding, Lina Wei, Lei Guo, Xiaozhou Sun, Lei Wang, Fushuang Yang, Liping Sun

**Affiliations:** a Changchun University of Chinese Medicine, Changchun City, Jilin Province, China; b Affiliated Hospital of Changchun University of Chinese Medicine, Changchun City, Jilin Province, China.

**Keywords:** meta-analysis, recurrent respiratory tract infections, Tuina

## Abstract

**Background:**

Recurrent respiratory tract infections (RRTIs) are common respiratory ailments in children. RRTIs are often difficult to control and thus generally have a long-term disease course. Children who receive ineffective treatments or those that experience poor treatment outcomes are prone to developing complications such as edema, cough and asthma. Such complications can seriously hinder a child's growth and development, while also adversely affecting the child's physical and mental health. Tuina massage, a traditional Chinese technique that has been practiced in China for >5000 years, has recently been used to treat RRTIs, with good effect. However, no systematic review of research studies focusing on massage as a treatment for RRTIs can be found in the literature to date. The purpose of this study will be to evaluate the efficacy and safety of Tuina massage for the treatment of pediatric patients who experience RRTIs.

**Methods:**

We will search the following databases using electronic methods: the Chinese Biomedical Literature Database (CBM), the China National Knowledge Infrastructure (CNKI), Wanfang Data (WAN FANG), VIP Information (VIP), MEDLINE, PUBMED, EMBASE, and CINAHL. For each database search, the scope will include articles published between the date of database inception to September 2021. Revman5.4 software will be used to conduct this systematic review and meta-analysis.

**Results:**

This meta-analysis will confirm whether Tuina massage is of clinical benefit to pediatric patients who experience RRTIs.

**Conclusion:**

The results of our systematic review and meta-analysis will be used to formulate conclusions as to whether massage therapy is an effective treatment for children suffering from RRTIs.

**Ethics and dissemination:**

This systematic review will evaluate the efficacy and safety of tuina in the treatment of recurrent respiratory tract infections. Since all the data included were published, the systematic review did not require ethical approval.

**INPLASY registration number:**

INPLASY202190107.

## 1 Introduction

Recurrent respiratory tract infections (RRTIs), which commonly afflict children, are diagnosed based on whether the frequency of upper and lower respiratory tract infections that occur during the course of 1 year exceeds the normal frequency.^[1]^ Frequent RRTIs can greatly affect a child's quality of life, while also leading to recurrent wheezing, malnutrition, anemia, growth retardation and even decreased lung function.^[2,3]^ In recent years, quantities of air pollution have increased in many regions around the world, resulting in long-term childhood exposure to fine particulates with diameters of 2.5 microns or less (PM2.5) that appear to be involved in the observed increase in incidence of childhood RRTIs.^[4]^

To date, research on RRTIs has mainly been focused on clinical symptomatic treatments based on current medical practices including the administration of immunomodulators and/or supplements (trace elements and vitamins), as well as on antibiotics and other anti-infective therapies for patients with signs of infection.^[5–8]^ Although such interventions can exert a certain curative effect by alleviating RRTI symptoms in the short term through regulation of certain physiological functions, these interventions do not prevent relapses, are expensive and often must be used long term. Therefore, cheaper and safer treatments for RRTIs are needed. Meanwhile, recent studies have shown that pediatric massage can significantly improve RRTI patient symptoms, reduce recurrence rates, and reduce incidence rates of adverse events.^[9]^

Tuina massage, a type of natural therapy based on traditional Chinese medicine, has been practiced in China for thousands of years. Massage therapies can be very effective when used to treat pediatric patients, since children have delicate skin and thus are highly sensitive to physical stimulation delivered in the form of massage. Moreover, children are able to easily receive and transmit signals generated through physical manipulations to relevant viscera, resulting in alteration of the visceral pathological state. Massage has been shown to effectively invigorate the positive qi and dispel the evil qi by adjusting the function of qi within the blood and viscera. In addition, massage has advantages that include painless delivery, a high level of patient acceptance and good curative effect.^[10]^ Furthermore, studies have shown that massage can regulate functions of immune system organs, cells and molecules. Taken together, results of previous studies indicate that massage administered within a clinical setting can serve as an effective intervention for treating children suffering from RRTIs.^[11,12]^ However, to the best of our knowledge no systematic review of pediatric massage therapy for RRITs has been published to date. Therefore, the purpose of this study is to evaluate the efficacy of infantile Tuina massage as a treatment for RRITs.

## 2 Methods and analysis

### 2.1 Design and registration information for the systematic review

This systematic review protocol has been registered with the International Platform of Registered Systematic Review and Meta-Analysis Protocols (INPLASY) and has been assigned the registration number INPLASY202190107.The study aims to evaluate the effectiveness and safety of Tuina for use in the treatment pediatric RRITs.

### 2.2 Inclusion criteria for study selection

#### 2.2.1 Types of studies

Only randomized controlled trials (RCTs) published or registered before September 1, 2021 are included.

#### 2.2.2 Types of participants

Pediatric patients with clear TTRI diagnosis.

#### 2.2.3 Types of interventions and comparators

Pediatric massage, unlimited manipulation; control measures: blank control, or any standard drug control supported by previous evidence, such as pidotimod, spleen aminopeptide oral lyophilized powder. or supplement containing trace elements and multivitamins. Studies of pediatric massage combined with other treatments and homeotherapies were also included.

#### 2.2.4 Types of outcomes

Primary prognostic indicators (outcomes) included curative effect and the number of episodes of respiratory tract infection, whereas secondary prognostic markers included levels of immunoglobulin (IgA, IgG, IgM), and T lymphocytes (CD3+, CD4+, CD8+, CD4+/CD8+).

### 2.3 Search methods to screen for relevant studies

Databases used for comprehensive literature searches will include the Chinese Biomedical Literature Database (CBM), the China National Knowledge Infrastructure (CNKI), Wanfang Data (WAN FANG), VIP Information (VIP), MEDLINE, PubMed, Excerpta Medica Database (EMBASE), and the Cumulative Index to Nursing and Allied Health Literature (CINAHL). Searching of each database will cover the time frame between the date of database inception and September 2021.We will also search for relevant minutes of meetings, test registers and established reference lists of publications for use in future searches.

The following group of search terms will be used for searches (“recurrent respiratory infections,” “massage” or “massage” and “RCT”). Subsequent searches will use medical topic titles (MESH), including “recurrent respiratory infections,” “massage,” and “massage,” as well as the keywords initially retrieved. Other article searches will review the list of references obtained using related research articles. For example, Table 1 summarizes the PubMed search strategy.

**Table 1:** Search strategy for PubMed.

No	Search terms
#1	“Massage”[MeSH] or“Massage Therapy”[Title/Abstract] or“Tuina”[Title/Abstract]
#2	“RRTIs”[Title/Abstract] or“recurrent respiratory tract infections”[Title/Abstract] or“respiratory tract infections”[Title/Abstract] or “RTI”[Title/Abstract]
#3	“Randomized controlled trial”[Title/Abstract] or“Controlled clinical trial”[Title/Abstract]
#4	#1 and #2 and #3

### 2.4 Study selection and data extraction

**Figure 1:**
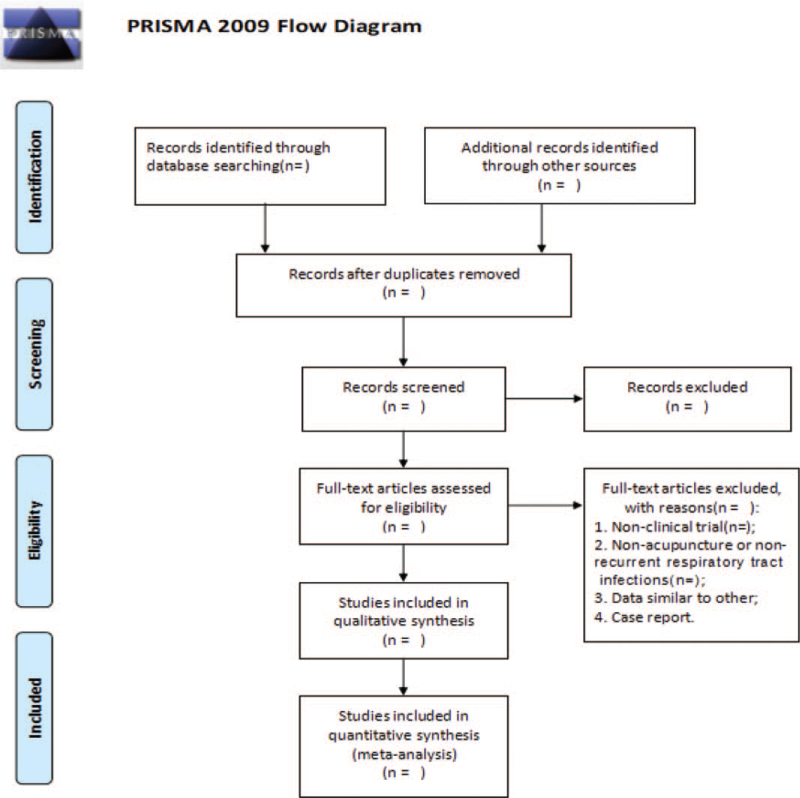
PRISMA flow diagram of study and exclusion. PRISMA, Preferred Reporting Items for Systematic Reviews and Meta analyses.

Two investigators (YT and LG) will independently review literature screening results and will perform data extraction. Literature screening will be performed according to the inclusion criteria used to identify appropriate studies, with disagreements resolved by discussion or mediated by a third party (ZW). The final selection process will adhere to Preferred Reporting Items for Systematic Reviews and Meta-Analyses (PRISMA) guidelines,^[13]^ as shown in Fig. 1.

Two reviewers will independently extract the data and complete the predefined data extraction form. This task will include entering information into the data extraction table as follows: basic information regarding the study (researcher's name, research title, year of publication, country, language, publication status); study characteristics (sample size, case source, age, course of disease, diagnostic criteria, inclusion criteria, exclusion criteria); intervention and control measures; research methodology (generation of random schemes, allocation concealment, blinding method, baseline comparability, loss to follow-up). Measurement data will be used to determine meta-analysis outcomes indices.

### 2.5 Risk of bias assessment

The Cochrane bias risk assessment tool^[14]^ will be used to evaluate the quality of the methodology used to search the literature. Because the subjects of this study are children under 18 years of age, main outcome indicators will include curative effect and frequency of respiratory tract infections, whereas immunoglobulin levels and T lymphocyte levels will serve as relative objective indicators. We think that implementation of a blinded method would have little effect on the outcome, since the intervention under evaluation in this meta-analysis is a non–drug-based therapy, making implementation of a placebo-controlled blinded study difficult. Thus, the term “blind method for study subjects” was removed from the original set of 7 search terms. Quality evaluation results for each item will be divided into three grades: “low bias risk,” “high bias risk” and “bias risk uncertainty.”

### 2.6 Missing data management.

We will contact the original authors of papers with missing data via email and will wait for a reply for 1 month after sending the email. If the missing data cannot be obtained, articles containing incomplete data will be excluded from the final analysis.

### 2.7 Statistical analysis

#### 2.7.1 Heterogeneity test and meta-analysis

Data will be subjected to statistical analysis using RevMan5.4 software. First, the results of a single study will be described. Next, relative risk (RR) and its 95% confidence interval will be used as dichotomous outcome variables of massage safety and efficacy; the mean difference and its 95% confidence interval will be used as continuous variables to describe massage effects as numerical values derived from inter-group comparisons. To assess clinical heterogeneity between studies, clinical heterogeneity of study subjects, intervention measures, control measures, and outcome indicators each will be assessed for similarities among the different studies. To assess statistical heterogeneity between studies, statistical heterogeneity will be judged according to the results of the *I*^2^ test; if *I*^2^ >75%, the statistical heterogeneity between studies is very significant but does not meet the level of significance required for meta-analysis studies; 25% ≤ *I*^2^ ≤75% indicates that clinical homogeneity is better than that mentioned above based on a random effect model; when *I*^2^ <25%, a fixed-effect model is used.

#### 2.7.2 Sensitivity analysis

Wherever possible, we will conduct sensitivity analysis to explore the impact of bias risk of a trial on the preliminary results. These analyses will exclude lower-quality trials, duplicates of meta-analysis papers, studies with insufficient sample size and/or insufficient data for assessing data quality and robustness when significant statistical heterogeneity is found.

#### 2.7.3 Assessment of publication biases

The funnel chart drawn by RevMan 5.3 software will be used to detect publication bias.

#### 2.7.4 Subgroup analysis

For robust data, subgroup analysis will be conducted using several different types of controls to control for “different pediatric massage techniques” and “different courses of treatment”. Next, the stability of the results will be judged using sensitivity analysis (only high-quality studies will be compared with the combined results of all studies).

#### 2.7.5 Grading the quality of evidence

The Grading of Recommendations Assessment, Development, and Evaluation (GRADE) quality evaluation method will be used to evaluate the quality of meta-analysis results regarding patient outcomes. Based on aspects of methodological quality, the consistency of the results among studies, the directness and accuracy of evidence, and the possibility of publication bias, we will judge whether the evidence level of results of RCTs should be degraded or not. As a final step, we will determine whether the evidence grade is high, medium, low, or very low.

## 3 Discussion

RRTIs commonly afflict children in increasing numbers each year, due to natural and man-made alterations of human living environments. At present, modern medical interventions to treat RRTIs are largely based on relieving clinical symptoms in combination with immunomodulators or dietary supplements containing trace elements and vitamins. Thus, some children mainly receive broad-spectrum antibacterial therapy, a practice that has led to a significant increase in bacterial drug resistance due to antibiotics misuse, resulting in decreased efficacy of these drugs when used to treat RRTIs. By contrast, massage is a traditional treatment method that works by dredging channels and collaterals to enhance immune system function and strengthen the physique. Pediatric massage combines specific manipulative techniques applied to acupoints throughout the body to therapeutically improve bodily functions. Although Tuina massage is viewed as a type of pure physiotherapy, its benefits overlap with benefits of modern medicines while also offering advantages of simple operation, lack of apparent adverse reactions, low price, among others. In recent years, the number of clinical trials investigating massage as a treatment for RRITs has increased, making it necessary to update systematic reviews of RRTIs. Thus, it is hoped that the results of this systematic meta-analysis-based study will provide a rationale for use of acupuncture in the form of Tuina massage for the treatment of RRTIs.

## Author contributions

**Conceptualization:** Ye Tian.

**Data curation:** Ye Tian, Lei Guo.

**Formal analysis:** Lie Wang, Zhongtian Wang, Xiaozhou Sun.

**Funding acquisition:** Liping Sun.

**Investigation:** Lizhong Ding, Lina Wei.

**Methodology:** Ye Tian.

**Supervision:** Liping Sun.

**Validation:** Lei Wang, Fushuang Yang.

**Visualization:** Ye Tian.

**Writing – original draft:** Ye Tian.

**Writing – review & editing:** Ye Tian, Lie Wang, Liping Sun.
